# Clinical research ethics review process in Lebanon: efficiency and functions of research ethics committees – results from a descriptive questionnaire-based study

**DOI:** 10.1186/s13063-017-2397-2

**Published:** 2018-01-11

**Authors:** David Atallah, Malak Moubarak, Nadine El Kassis, Sara Abboud

**Affiliations:** 1 Department of Gynecology and Obstetrics, Hôtel-Dieu de France University Hospital, Beirut, Lebanon; 2 Faculty of Medicine, Saint Joseph University, Beirut, Lebanon; 3 Health Department, University of Liverpool, Liverpool, UK

**Keywords:** Adherence, Effectiveness, Performance, Research ethics committees, Scores

## Abstract

**Background:**

Clinical trials conducted in Lebanon are increasing. However, little is known about the performance of research ethics committees (RECs) in charge of reviewing the research protocols. This study aimed to assess the level of adherence to the ethics surrounding the conduct of clinical trials and perceptions of team members regarding roles of the RECs during the conduct of clinical trials in Lebanon. The research question was: Are RECs adherent to the ethics surrounding the conduct of clinical trials (chapters II and IV in ‘Standards and Operational Guidance for Ethics Review of Health-related Research with Human Participants’ in Lebanon?’

**Methods:**

This was a quantitative and descriptive questionnaire-based study conducted among RECs of university hospitals in Lebanon. The questionnaire had to be completed online and included general questions in addition to items reflecting the different aspects of a REC performance and effectiveness. All the questionnaire was assigned a total score of 175 points. General information and questions assigned point values/scores were analysed using descriptive statistics: frequency and percentage, mean score ± standard deviation.

**Results:**

Ten RECs participated in the study (52 persons: four chairs, one vice-president, 47 ordinary members). Forty-seven (90.4%) had previous experience with clinical research and 30 (57.7%) had a diploma or had done a training in research ethics. Forty-one percent confirmed that they were required to have a training in research ethics. All RECs had a policy for disclosing and managing potential conflicts of interest for its members, but 71.8% of participants reported the existence of such a policy for researchers. Thirty-three point three percent reported that the RECs had an anti-bribery policy. The questionnaire mean score was 129.6 ± 22.3/175 points reflecting thus an excellent adherence to international standards.

**Conclusion:**

Inadequate training of REC members and the lack of anti-bribery policies should be resolved to improve their performance.

**Electronic supplementary material:**

The online version of this article (doi:10.1186/s13063-017-2397-2) contains supplementary material, which is available to authorized users.

## Background

Medical and clinical research activities involving human subjects are being increasingly performed in the developing countries [1]. For instance, the number of clinical studies increased by 30% from 167 to 217 trials (phases 1 to 4) in Lebanon between April 2013 and May 2017 [2, 3]. This growth is due to many factors such as the availability of clinical research units and clinical research organisations dedicated solely to clinical research, in addition to the huge investment in healthcare expenditure. Other factors include the presence of treatment-naïve populations and the economic status of many patients who do not have social security coverage or access to high-quality medical care. Thus, participating in clinical trials would afford them their treatment. In parallel, new ethical standards for performing such studies have been released by international organisations such as the ‘Ethics of Research Related to Healthcare in Developing Countries’ by the Nuffield Council on Bioethics in 2002 [4]; the ‘International Ethical Guidelines for Biomedical Research Involving Human Subjects’ by the Council for International Organisations of Medical Sciences (CIOMS) in 2002 in collaboration with the World Health Organisation (WHO) [5]; and the latest amendment of the Declaration of Helsinki by the World Medical Association (WMA) during its 64th general assembly in 2013 [6]. Despite the availability of ethical guidelines, study investigators working in the developing world including the Middle East region, are confronted with issues and doubts concerning the ethical validity of clinical research and the capacity of research ethics [4, 7]. In this context and on a global scale, Miller reported in his case analysis of 2002 that the ethical conduct of clinical trials sponsored by the pharmaceutical companies is raising few concerns. Such concerns are focussed on the scientific quality of these trials and the extent to which the trials’ participants are adequately protected. Importantly, these concerns are likely to be related to the sponsorship of these trials by the pharmaceutical companies and their conduct by community physicians [8]. Okike [9] also states that financial conflicts of interest are also problematic in clinical research as positive conclusions are often more likely to be made by investigators with conflict of interests. He explains that such bias might be linked to biased study design or biased explanation of the trials’ results by the investigators, or deletion of negative results by the pharmaceutical companies. Other factors include ‘preferential funding by industry of projects that are likely to succeed’ [9].

In Lebanon, scientific research and clinical trials involving human participants are currently performed in research and medical centres. Also, the number of pharma sponsors involved in clinical research in the country is increasing [10]. This situation behoved the National Council for Scientific Research (NCSR) to publish the ‘Charter of Ethics and Guiding Principles of Scientific Research’ in July 2016 [11]. However, little is known about the performance of the ethics review system and the adherence of research ethics committees (RECs) to this charter and international ethical guidelines and their level of independence from the pharmaceutical companies. Also, the Lebanese guidelines regarding the ethical protection of human subjects participating in clinical trials has only two legal protections [12], which is the lowest number in the Arab world. Indeed, the Qatari guideline has the highest number of protections (19 protections), followed by the Saudi ‘Clinical Trial Requirement Guidelines’ (15 protections) [13]. Moreover, little is known about the adherence of RECs in Lebanon to the ‘Standards and Operational Guidance for Ethics Review of Health-related Research with Human Participants’, which were developed by the WHO in 2011 [14]. This document consists of 10 standards/principles/norms to be followed by the RECs while reviewing the ethical aspects of health-related research activities involving humans. These standards do not suggest new concepts for REC functioning nor in resolving specific ethical dilemmas. They do not aim to replace the existing laws nor the necessity for developing local guidelines for the ethical review of studies conducted among humans. Instead, they refer to requirements for RECs outlined in prevailing international guidelines, bring attention to the ethical review of research, complement existing regulations and laws, and allow RECs to develop their own practices and written procedures. Ultimately, adherence to these 10 standards would help the RECs to reach high-quality performance [14].

Given these considerations, it seemed important to present an in-depth assessment of RECs in Lebanon, their adherence to chapters II and IV of the ‘Standards and Operational Guidance for Ethics Review of Health-related Research with Human Participants’ [14], and the RECs members’ perceptions regarding roles of the RECs during the conduct of clinical trials in Lebanon. Chapter II (standards 2 to 6) is mainly focussed on the functions of RECs, members’ composition and training, their independence, and any potential conflicts of interests, while chapter IV (standard 9) delineates ‘guidance for the secretariat staff, and administration of the RECs’ [14].

## Methods

### Research question and study objectives

The research question was: ‘Are RECs adherent to the ethics surrounding the conduct of clinical trials (chapters II and IV in ‘Standards and Operational Guidance for Ethics Review of Health-related Research with Human Participants’ [14] in Lebanon?’ Thus, the study objectives were to describe the qualifications and education of the members of these RECs; to describe policies in place to address the education of REC members, and to describe the adherence of these RECs to World Health Organisation (WHO) ‘Standards and Operational Guidance for Ethics Review of Health-related Research with Human Participants’.

### Study design

This was a quantitative and descriptive questionnaire-based study (survey), conducted among RECs of university hospitals in Lebanon.

### Sample size

All members of RECs affiliated to university hospitals in Lebanon were to be included in this study. As of 9 January 2017, a list of university hospitals in Lebanon has been established (*n* = 28). The total sample size was estimated to be 280 participants as each REC of the 28 RECs in Lebanon has an average of 10 members. Response rate was estimated to reach 30% (*n* = 84) based on local colleagues’ experience with surveys (no supportive academic literature data). However, a list of authorised RECs in Lebanon was provided in March 2017 by the Lebanese Ministry of Public Health showing that 12 RECs were authorised by October 2016 for 3 years in the country. The updated and final sample size would account for 123 members instead of 280, as per the new list, and the final number of respondents is expected to reach 37 participants (chairs and members).

### Sample recruitment and setting

The questionnaire was first prepared on papers (Additional file 1) and reviewed by the dissertation advisor at the University of Liverpool. The questionnaire was then transcribed online using SurveyMonkey® (SurveyMonkey Inc., San Mateo, CA, USA, www.surveymonkey.com), and tested for feasibility before being sent to the participants. Afterwards, an email was sent separately to each president/chair and all members of the selected RECs inviting them to answer the questionnaire. The email text consisted of the Participant Information Sheet including the link to the study questionnaire to be completed online. The questionnaire completion was equivalent to consenting to participate in the study. A reminder was sent by email in case the questionnaire was not returned back within 15 working days. If no questionnaire was returned back within seven working days, a phone call was made to the concerned person asking them to complete the questionnaire, provided that they accepted to participate in the study. No follow-up was made with the persons who did not agree to participate in the study. Each participant was also allocated a participant identification number. A pilot study was conducted to assess the feasibility of the study (two participants). In case the respondents faced issues in completing the questionnaire, based on their feedback, a shorter version of the document was prepared before re-sending to all participants.

### Data collection methods

Data were collected through an online questionnaire to be completed by the presidents/chairs and all members of all RECs in Lebanon. The questionnaire was derived from the ‘Research Ethics Committee Quality Assurance Self-assessment Tool’ developed by Sleem et al. in 2010, and where each item was assigned a point value or a score with a maximum score of 200 points for the entire questionnaire [15]. Of note, a maximum score of 175 points was assigned to the modified version of the questionnaire (Additional file 1) versus 200 points to the original questionnaire. Also, the two topics about the workload of REC (score of 0 points in the original version) and minutes (score of 13 points in the original questionnaire) were deleted in the modified version. The two additional topics covered by the modified version of the questionnaire (general information and conflicts of interests/anti-bribery were assigned a score of 0 points (Table 1).

**Table 1:** Comparison between the original and modified version of ‘Research Ethics Committee Quality Assurance Self-Assessment Tool’

	Original version (Sleem et al., 2010) [15]	Modified version (Present study)
Number of topics	10	10
Total number of questions	181	110
Number of questions by topic (maximum number of points by topic)		
(1) General information	0 (–)	9 (zero)
(2) Organisational aspects	15 (54)	15 (54)
(3) Conflicts of interests/anti-bribery/anti-corruption policy	0 (–)	3 (zero)
(4) Membership and educational training	9 (30)	9 (30)
(5) Submission arrangements and materials	12 (12)	7 (7)
(6) Minutes	9 (13)	0 (–)
(7) Policies referring to review procedures	11 (11)	9 (9)
(8) Review of specific protocol items	44 (43)	38 (38)
(9) Communicating a decision (approval letter)	5 (5)	5 (5)
(10) Continuing review	12 (16)	12 (16)
(11) REC resources	3 (16)	3 (16)
(12) Workload of the REC	11 (0)	0 (–)
Maximum score (points)	200	175

This questionnaire developed by Sleem et al. [15] is notably easy to be completed. Based on international ethics guidelines, it investigates the protection of human subjects involved in clinical studies, and the administrative procedures adapted by many RECs in the developing world. The total score reflects the REC performance limitations, such as any REC strategies, against recognised international standards. The higher the score, the fewer are the limitations A high score provides positive feedback on the effectiveness of RECs. The score’s interpretation is suggested in Table 2; it is not derived statistically but rather based on a pragmatic and personal opinion in the absence of supporting materials.

**Table 2:** Questionnaire’s score interpretation

Total score (points) Original questionnaire (Sleem et al., 2010) [15]	Total score (points) Modified questionnaire (present study)	Interpretation
>130/200	>114/175	Excellent adherence
66–130/200	58–114/175	Good adherence
>66/200	>58/175	Poor adherence

### Ethical considerations

Conducted among adult members of RECs in Lebanon, this study involves human subjects. However, no benefits or hazards are discerned in this context. Thus, no approval was required from any local ethical committee in Lebanon, except from one university medical centre in Beirut, Lebanon. In parallel, the study could have had an expedited review because there were limited risks, and the present proposal was submitted to the University of Liverpool in February 2017 for ethics clearance, which was given in March 2017.

### Analytical approach

For each REC member who completed the questionnaire, general information and questions that were assigned scores were analysed using descriptive statistics: numbers and percentages for categorical data, means and standards deviations for continuous variables. No replacement for missing data was performed. Given that the present study was descriptive and exploratory where data are collected to see where they lead, no prior hypothesis was set and *p* values were not required. Given these considerations, inferential statistics were not performed. The survey data were exported from SurveyMonkey® to an offline computer database, and data were analysed anonymously using IBM SPSS, version 24.0 for Windows Release (IBM Corp. Released 2016. IBM SPSS Statistics for Windows, Version 24.0. Armonk, NY, USA: IBM Corp.).

## Results

After testing the online questionnaire for feasibility before being sent to the participants, no modifications were made to the questionnaire, and data were collected over 2 months from 8 April 2017 to 1 June 2017. The study database was cleaned and locked on 2 June 2017.

### Number and response rates of participating RECs, chairs, and members

**Fig. 1:**
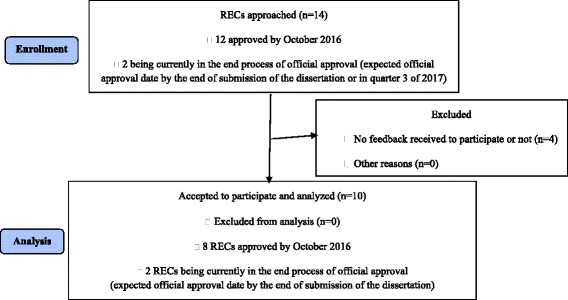
Flow diagram of participating research ethics committees (RECs)

In total, 14 RECs were approached of which two (five members) RECs were currently in the final process of official approval by the Ministry of Public Health in Lebanon. These two RECs had received non-official approval by the time that they were approached for study participation and they will be officially declared as approved RECs in the third quarter of 2017. Out of the 12 RECs that were officially authorised by October 2016 for 3 years in Lebanon by the Lebanese Ministry of Public Health and who were approached to participate in the study, 8 (66.8%) RECs agreed to participate, while 4 (33.2%) RECs had not expressed any positive or negative will to participate in the study by 1 June 2017. In addition, the two RECs in the final process of official approval participated in the study. Thus, a total of 10 RECs completed the study questionnaire (Fig. 1).

The total number of chairs, vice-presidents, and ordinary members from the 10 participating RECs was 103 persons. Fifty-two out of 103 persons, including chairs and members of the 10 RECs, completed the online questionnaire, accounting thus for a total response rate of 50.5%, which is higher than the estimated response rate of 30% in the ‘Sample size’ section above. The minimal response rate was 20% (one respondent of five) in one of the participating RECs, and the maximal response rate reached 100% in two other Lebanese RECs. The mean number of respondents was 5 ± 4 persons per REC (minimum 1; maximum 13) (Table 3).

**Table 3:** Response rate by participating REC and for all RECs

REC	Number of respondents	Total number of chairs, vice-presidents and member	Response rate
REC 1	13	13	100%
REC 2	8	15	53.3%
REC 3	4	11	36.4%
REC 4	3	12	25%
REC 5	3	18	16.7%
REC 6	5	9	55.5%
REC 7	10	10	100%
REC 8	1	5	20%
REC 9	2	5	40%
REC 10	3	5	60%
Total	52	103	50.5%

### Position of the participants, distribution of participants by geographical area of the affiliated hospital, and year of establishment of the RECs

Out of the 52 participants, 4 (7.7%) persons were presidents or chairs of RECs, 1 (1.9%) participant was vice-president of an REC and 47 (90.4%) were members of RECs. Most of the participants were affiliated to RECs of university hospitals located in Beirut: 41 (78.8%) persons, and the other 11 (21.2%) participants were affiliated to RECs of university hospitals located in Mount Lebanon. The median year of the establishment of the participating Lebanese RECs was 2001 (Q1 = 2000; Q3 = 2010). On average, the 10 participating RECs were established in 2004 ± 6.8 (minimum 1994; maximum 2016).

### General information: participants’ education characteristics and affiliations with the REC’s institution

Out of the 52 participants, 34 (65.4%) persons had a medical education, 14 (26.9%) persons had a non-medical and scientific education, and 4 (7.7%) persons had a non-scientific education. Also, 31 (59.6%) participants had a medical (MD) degree, 11 (21.2%) had a Doctor of Philosophy (PhD) degree, 5 (9.6%) had a Master of Science (MSc) degree, and 11 (21.2%) persons had other academic qualifications such as nursing, law, and dentistry degrees. As an aside, some of the participants had at least one academic degree. The majority (47 (90.4%)) of the participants had previous experience with clinical research while 5 (9.6%) had none. Moreover, 30 (57.7%) participants held a diploma or had trained in medical and/or research ethics. Forty-three (82.7%) participants were affiliated with the REC’s institution.

### Distribution of REC members who completed all the items of the questionnaire and missing data

Out of the 52 participants, 13 (25%) persons did not complete the full questionnaire. Thus, the statistical analysis of the scores and the questionnaire’s topics (excluding the general information) was performed on the 39 (75%) participants who completed the entire questionnaire. Missing date were due to incomplete answers from regular members. All chairs (*n* = 4, 100%) and vice-presidents (*n* = 1, 100%) completed all the questions. Thus, the following sections describe the results from the 39 participants who completed all the questionnaire’s items.

### Organisational aspects

Out of 39 respondents, 37 (94.9%) participants reported that the REC is subject to registration with a national authority versus 2 (5.1%) participants who reported the opposite. Twenty-seven (69.2%) persons replied that the REC meets once per month as a full committee to review the research studies while 9 (23.1%) persons said that the meetings are held every 2 months and 3 (7.7%) twice per month. Only 1 (2.6%) person added that the meetings are held upon request. All of the 39 (100%) participants confirmed that the REC was established under a high-ranking authority, that it has written Standard Operating Procedures (SOPs) and a policy for disclosing and managing potential conflicts of interest for its members. However, the availability of a policy for disclosing and managing potential conflicts of interest for members of the research team was reported by 28 (71.8%) persons.

Thirty-one (79.5%) participants replied that the REC has a policy that summarises the process for appointing the REC chair. As for the criteria used to select the chair of the REC, prior research experience was chosen by 27 (69.2%) persons, prior training in ethics by 21 (53.8%) participants, and publication in ethics by 2 (5.1%) persons. Eight persons each chose one of the following criteria: appointed by the chairman of the hospital; criteria of the Ministry of Public Health; institutions regulations; being the Director General of the hospital; being nominated by the Dean of the Faculty of Medicine and Medical Sciences; professional experience and academic qualifications; being religious and a director of the hospital; and status of the university hospital.

Furthermore, 35 (89.7%) participants answered that the REC has a policy that defines the procedure for assigning the REC’s members and provides details on the membership requirements and the appointment conditions. To be selected as a member of the REC, 29 (74.4%) persons reported that prior training in ethics and prior research experience are used as criteria in their institution, followed by publication in ethics (10 (25.6%) persons). In addition, 13 (33.3%) participants stated the following criteria: appointed according to the status of the hospital; criteria of the Ministry of Public Health (e.g. multidisciplinary team and specialisation fields); clinical activity of the members; faculty members representing different schools; members of the civil society from different relevant fields; nominated by the Medical Director of the hospital; professional curriculum vitae (CV) and academic qualifications; social work; and having one of these academic qualifications: MD, MD with training in research, a fellow, an ethicist, or a lawyer.

Less than the half of the participants (14 (35.9%) participants) said that a quality programme for the REC is available. The major activities that were undertaken in the last year and the changes made because of the quality improvement programme were namely:

1. Continuous improvement and awareness sessions: 1 (2.6%) respondent
2. Mandatory online ethics course of the National Institute of Health (NIH) and examination on medical research for the REC members + series on medical research done frequently and regularly at the institution: 2 (5.2%) respondents
3. Training on research methodology for all members + mandatory Collaborative IRB Training Initiative (CITI) examination for all members and all persons conducting research: 1 (2.6%) respondent
4. Lectures and workshops organised to provide training to members of an REC accredited by the Joint Commission International, and to other personnel in the medical field on proper conduction of research: 1 (2.6%) respondent
5. Hosting annual surveys soliciting feedback from the community on their knowledge of and experience with the processes administered by the REC: 1 (2.6%) respondent
6. Ongoing procedure: 3 (7.8%) respondents

Thirty-one (79.5%) respondents said that the institution/organisation regularly evaluates the operations of the REC (e.g. financial needs, appropriateness of material resources, policies, procedures, practices, and membership given the study being reviewed, and documentation of the training requirements of the REC members). In parallel, 34 (87.2%) participants answered that the REC has a process whereby enrolled research participants can file complaints or direct questions concerning issues about the human subjects’ protection. The mechanisms are described in Table 4.

**Table 4:** Mechanisms whereby enrolled research participants can file complaints or direct questions regarding human subjects’ protection issues (*N* = 17)

	Number (n)	Percentage (%)
A letter + file or a complaint form addressed to the research department and/or REC (hard or soft copies).	6	35.4
Participants sign an informed consent which details the contact information of the principal investigator and the REC in case they want to contact them.	5	29.4
Multiple ways by which members or the chair of the REC may be contacted for clarification on decisions or on policies are clearly listed on the REC’s website.	2	11.8
Phone call or letter sent to the secretariat of the REC.	2	11.8
An interview between the committee and research participants.	1	5.8
A formal letter sent to the chair of the committee to be discussed in the meeting for recommendations.	1	5.8

Twenty-three (59.0%) participants reported that the records of the REC are stored electronically in a secured computer protected with a password, while 15 (38.5%) answered that records are stored in paper folders in a locked file cabinet. Only 1 (2.6%) person reported another filing system without describing it. The majority of the participants (36 (92.3%) persons) confirmed that the REC requires a quorum to make the meeting official and review the study protocols.

### Conflict of interests/anti-bribery/anti-corruption policy

Less than 50% of the participants (13 (33.3%) persons) reported that the REC has an anti-bribery/anti-corruption policy, and only 3 (7.7%) confirmed having a policy that defines whether the members have to be trained on an anti-bribery and anti-corruption course. The training was reported to occur when a new member joins the REC. Only 1 (2.6%) participant answered that they had accepted goods because it is difficult to refuse gifts from some patients who might consider refusing a gift to be an insult. However, they had never accepted money.

### Membership and educational training

Most of the RECs consisted of 12 (38.5%) members. Two point six percent of RECs were composed of eight members and 5.1% of RECs consisted of nine members. Thirty-eight point five percent of the participants said that the RECs were constituted of nine men and 23.1% of nine women, also 10.3% reported that the RECs were composed of eight men and 2.6% stated that the RECs were composed of eight women. On average, the RECs were composed of more men (average of 8 ± 2 men per REC; minimum 3; maximum 10) than women (5 ± 3 women per REC; minimum 1; maximum 9) with a mean female/male ratio of 0.625.

Only 1 (2.6%) participant replied that the REC has a member not affiliated with the institution, and 37 (94.9%) participants said that some of the REC members are considered to be non-scientists. Twenty-two (56.4%) participants reported that the REC requires that its chair should have a prior formal training in research ethics: web-based training (*n* = 12, 30.8%), workshop in research ethics (*n* = 12, 30.8%), courses (*n* = 8, 20.5%), CITI training (*n* = 1, 2.6%), and spiritual training for religious chairs, since 2 (5.2%) participants reported that the directors of the RECs are a sister or a priest. Moreover, 16 (41.0%) persons confirmed that the institution requires from REC members to have training in research ethics to be a member of the REC: web-based training (*n* = 12, 30.8%), workshop in research ethics (*n* = 12, 30.8%) in addition to experience (*n* = 1, 2.6%). Similarly, 16 (41.0%) persons confirmed that the institution requires that investigators have training in research ethics to submit protocols to be reviewed by the REC: web-based training (*n* = 12, 30.8%), workshop in research ethics (*n* = 4, 10.3%), lecture (*n* = 4, 10.3%), and courses (*n* = 5, 12.8%).

As for continuing education in research ethics, 18 (46.2%) persons answered that the REC conduct it for its members on a regular basis. Also, 27 (69.2%) participants declared that the trainings about human subjects’ protection received by the REC members are documented by the committee.

### Submission arrangements and materials

**Fig. 2:**
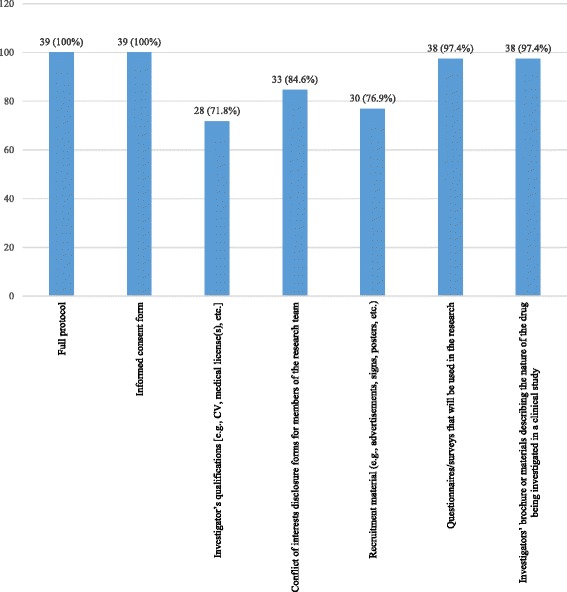
Submission materials requested from the Principal Investigators when they submit their research protocol to the REC (*N* = 39)

Regarding submission arrangements and materials, all the 39 (100%) respondents answered that a full study protocol is requested from the principal investigators when they submit their research protocol to the REC (Fig. 2).

### Policies referring to review procedures

**Fig. 3:**
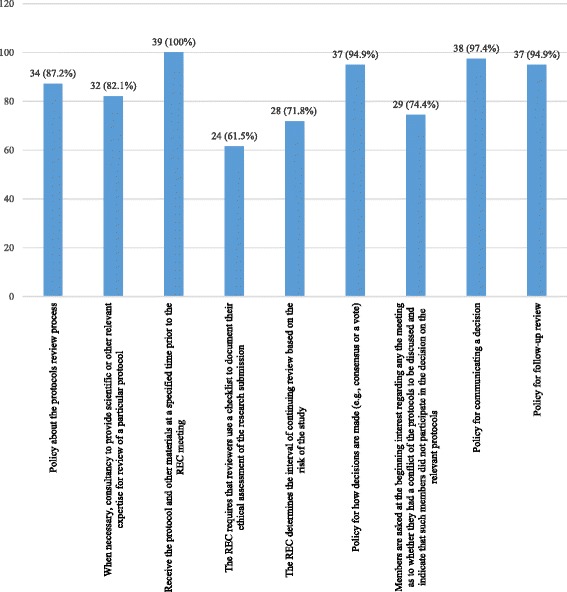
Existence of policies referring to review procedures (*N* = 39)

Out of the 39 respondents, 34 (87.2%) persons confirmed the availability of a policy for reviewing protocols and 32 (82.1%) persons reported that the REC brings in a consultant when deemed necessary to review a specific protocol (Fig. 3).

### Review of specific protocol items

Twenty-eight (71.8%) respondents answered that the REC reviews the appropriateness of the investigators’ qualifications to conduct the study. Also, more than 90% of them confirmed that the REC reviews the protocol items related to the considerations of risks and benefits, selection of research participants, privacy and confidentiality, community consultation, safety monitoring and adequacy of insurance to cover research-related injury, and need for a child’s assent. As for the informed consent, 38 (97.4%) respondents confirmed that the REC reviews the process for obtaining informed consent, 27 (69.2%) reported that the REC waives the requirements to obtain informed consent that is based on written criteria. Also, RECs were reported to evaluate the basic elements of informed consent by all respondents, stating that the study involves research, explaining the research objectives, defining the expected duration of the person’s participation, describing the processes to be followed, and identifying any experimental procedures, etc. Thirty-seven (94.9%) participants indicated that the REC reviews whether the informed consent clarifies the availability of any medical treatments in case of injury and, if so, what the treatments consist of or, where further information may be acquired, for research involving more than minimal risk.

### Communication a decision (approval letter)

This section of the questionnaire was skipped by six participants since no approval letter was sent to the investigator. Among the remaining 33 participants, the approval letter was reported to provide a termination date that is 1 year from the date of the convened REC meeting in which the study was approved by 19 (57.6%) persons, and to require the investigators to submit to the REC any changes that take place in the research plan, as an amendment, by 32 (97.0%) persons. Also, the letter was described as requiring the investigators to quickly report to the REC any adverse events or unanticipated issues, by 30 (90.9%) persons, and any protocol deviations, by 31 (79.5%) persons. Twenty-two (56.4%) participants replied that the letter requires the investigators to use the REC-approved Informed Consent Form that is stamped with an expiry date.

### Continuing review

The results showed that 36 (92.3%) of the 39 respondents indicated that the REC demands a continuing review report from the investigators at least once per year (Table 5).

**Table 5:** Items requested in the continuing review report

	Number (n)	Percentage (%)
Number of persons enrolled	35	89.7
Gender and ethnic/religious breakdown of enrolled persons	24	61.5
Number of persons withdrawn from the research by the investigators	35	89.7
Withdrawal reasons	34	87.2
Number of persons who dropped out of the research	34	87.2
Reasons why persons dropped out	32	82.1
Verification that all persons gave their informed consent and that all signed consent forms are on file	31	79.5
Number and description of serious adverse events in the previous year	36	92.3
List of any protocol violations or deviations	34	87.2
Any safety monitoring reports	36	92.3
If the study is finalised, submission of a final report describing the study outcomes	36	92.3

### REC resources

RECs were reported to have their own yearly budget by 26 (66.7) respondents, and 18 (46.2%) stated that a budget is available for training REC members and administrative staff. The results for the physical resources of the REC are as follows: access to a meeting room: 39 (100%) persons; access to a computer and printer: 34 (87.2%) persons; access to the Internet: 33 (84.6%) persons; access to a facsimile: 18 (46.2%) persons; and access to cabinets for storage of the protocol files: 28 (71.8%) persons. Finally, 38 (97.4%) persons confirmed that the RECs have administrative staff assigned to them.

### Scores

The total mean score was 129.6 ± 22.3 points over 175 points for the 39 participants, reflecting thus an excellent adherence to chapters II (standards 2 to 6) and IV (standard 9) of WHO guidelines. The mean scores and subscores are available in Table 6 for all REC members.

**Table 6:** Scores and subscores

		39 participants who completed the 110 questionnaire’s items	4 chairs of RECs	34 ordinary members of RECs	1 vice-president
Overall score					
Mean ± standard deviation (points over 175)		129.6 ± 22.3	134.5 ± 15.8	129.2 ± 23.4	124
Median (points over 175)		133	137	133	–
Minimum; maximum		54; 158	113; 151	54; 158	–
Score interpretation: adherence to chapters II and IV of the ‘Standards and Operational Guidance for Ethics Review of Health-Related Research with Human Participants’ [14]		Excellent adherence	Excellent adherence	Excellent adherence	Excellent adherence
Subscores					
Organisational aspects	Mean ± standard deviation (points over 54)	33.4 ± 7.5	33.6 ± 6.0	33.4 ± 7.9	30
	Median (points over 54)	35	36	35	–
	Minimum; maximum	16; 43	25; 38	16; 43	–
Membership and educational training	Mean ± standard deviation (points over 30)	16.6 ± 7.6	18.0 ± 7.7	16.6 ± 7.8.	13
	Median (points over 30)	16	16.5	16	–
	Minimum; maximum	6; 28	11; 28	6; 28	–
Submission arrangements and materials	Mean ± standard deviation (points over 7)	6.3 ± 0.9	7.0 ± 0.0	6.2 ± 1.0	6
	Median (points over 7)	7	7	6.5	–
	Minimum; maximum	4; 7	7; 7	4; 7	–
Policies referring to review procedures	Mean ± standard deviation (points over 9)	7.6 ± 1.7	8.3 ± 1.0	7.6 ± 1.8	7
	Median (points over 9)	8	8.5	8	–
	Minimum; maximum	2; 9	7; 9	2; 9	–
Review of specific protocol items	Mean ± standard deviation (points over 38)	35.6 ± 3.6	35.8 ± 1.7	35.7 ± 3.9	34
	Median (points over 38)	36	35.5	36	–
	Minimum; maximum	16; 38	34; 38	16; 38	–
Communicating a decision (approval letter)	Mean ± standard deviation (points over 5)	4.1 ± 1.3	4.0 ± 1.0	4.1 ± 1.3	3
	Median (points over 5)	5	4	4	–
	Minimum; maximum	0; 5	3; 5	0; 5	–
Continuing review	Mean ± standard deviation (points over 16)	14.0 ± 4.0	14.0 ± 4.0	13.9 ± 4.2	15
	Median (points over 16)	16	16	16	–
	Minimum; maximum	0; 16	0; 16	0; 16	–
REC resources	Mean ± standard deviation (points over 16)	12.6 ± 3.5	15.3 ± 1.5	12.4 ± 3.6	16
	Median (points over 16)	14	16	14	–
	Minimum; maximum	1; 16	13; 16	1; 16	–

## Discussion

### Academic literature review

Social values must be incorporated in ethical clinical research to allow the expansion of knowledge and improvements in health. Nonetheless, translating results of clinical research into health improvements is random and complex [16]. With 80% of the world’s population living in the developing countries, an urgent need for clinical trials exists to help to report the huge burden of communicable and non-communicable diseases that they carry [4]. The ethics of clinical research conducted in developing countries like Lebanon are debatable [17–21]. Generally, three issues are the centres of these controversies. First, what are the standard of care to be used in research activities in developing countries? Second, what is the availability of interventions that are recognised to be beneficial during the research trials? Third, what is the quality of informed consent? [22]. Essentially, while medical research has to be ethically acceptable, the relevant information must be given to the potential participants in an intelligible way, and who must freely consent to participate without any obligation. Consenting is mainly vital in developing countries where many subjects give their informed consent as they believe that the clinical trial is their only way to receive healthcare, maybe because of financial constraints or an inefficient health system. In some developing countries, consenting might be done in an ineffective or inappropriate way due to differences in cultural and social backgrounds. In some cases, refusing participation in a trial is not possible because the person’s guardians or family members or any legal representative have assented to their participation. In other cases, persons may feel that participation in a clinical trial would allow them to ask questions during a meeting with the trial staff more than they could do in routine medical consultation [4].

The Working Party of the Nuffield Council on Bioethics recognises that clinical trials carried out in the developing world and financed by sponsors from the developed countries (termed externally sponsored research) must often face miscellaneous and sometimes contradictory regulations or guidance ‘as to what may be ethically acceptable’ [4]. In their report, which reviewed the ethics of a research project in the developing countries, the Working Party concluded that a system should be created in the developing countries to protect the human subjects participating in the clinical trials. Hence, they recommend ‘that all countries should establish an effective system for the ethical review of research, which includes the establishment and maintenance of RECs that are independent of government and sponsors of research’ ([4] p. 12).

Moreover, the Salim El-Hoss Bioethics and Professionalism Programme (SHBPP) (American University of Beirut, Faculty of Medicine, Beirut, Lebanon), discussed in September 2011 the results of a study aiming to assess the feasibility and potential usefulness of a self-assessment instrument [23]. The latter was a questionnaire developed by Sleem et al. in 2010 to evaluate the operations and effectiveness of RECs in the developing countries, given the unknown quality of the ethics review systems in these countries [15, 23]. The self-assessment questionnaire was composed of 181 questions covering 10 topics, namely: (1) organisational aspects (15 questions), (2) membership and educational training (9 questions), (3) submission arrangements and materials (12 questions), (4) minutes (9 questions), (5) policies referring to review procedures (11 questions), (6) review of specific sections of the study protocol (44 questions), (7) communicating a decision (approval letter) (5 questions), (8) continuing review (12 questions), (9) REC resources (3 questions), and (10) workload of the REC (11 questions) [15]. These elements were chosen by the authors since they reflect important characteristics of effective REC functioning, then reviewed and validated by bioethics experts and chairpersons of RECs in Sudan and Egypt. The elements’ choice was made on the basis that they would serve as indicators to appraise the efficiency of REC performance with regard to the protection of the human rights and the wellbeing of humans participating in medical research [15]. A point value or a score was attributed to each question with a maximum score of 200 points for the overall questionnaire [23]. The questionnaire was sent online to REC chairs in India, the Middle East, South Africa, Central and Eastern Europe [23]. The study showed that 36 chairs of RECs in this part of the world completed the self-assessment questionnaire: 91.7% of RECs had SOPs and 31.6% of them were founded under a high-ranking authority. In terms of policies, 80.6% of the participating RECs had a policy for the conflicts of interest and 27.8% had in place a programme for quality improvement. As for the education of the RECs members, 47.2% of these committees required their members to be trained in medical ethics, and continuing education for the RECs members was organised by half of the participating RECs. Additionally, 94.4% required a quorum, meetings were held at least once per month by 69.5% of the RECs, and 86.1% took minutes of meetings. As for the REC resources, almost one third of them (33.3%) had their own budget [23]. Hence, the SHBPP and the self-assessment tool provided RECs a means of reviewing their policies and processes versus the international standards [23].

As an aside, SHBPP was launched in April 2010 as an interdisciplinary support for parties involved in research, education, and consultation in bioethics in Lebanon and neighbouring countries, such as university instructors, students, policy-makers, and healthcare providers. It mainly seeks to improve public awareness, understanding, and academic activities on modern topics in bioethics. Essentially, SHBPP aims to improve the necessary skills and performances among medical staff who deal with issues like end-of-life care, law, policy, ethics, and research in Lebanon and the region [24].

Importantly, studies were able to identify many challenges hindering the effective functioning of RECs in the developing world, including Egypt, such as the limited financial and administrative resources of the RECs, non-sufficient or inadequate formal training of members and the lack of diversity of the member’s educational backgrounds [15, 25–28]. In addition, our search identified many studies highlighting the deficiency in ethics performance in the Middle East [29, 30]. Also, the lack of regulatory guidance was perceived as challenging by 92% of the participants in an Egyptian study [13]. The same study identified six out of 13 Middle Eastern countries that have their own national guidelines about ethical considerations in clinical research (Bahrain, Jordan, Kuwait, Qatar, Saudi Arabia, and the United Arab Emirates) versus Egypt and Lebanon which ethical guidance is referred to in medical professional guidelines [13]. Outstandingly, Yemen has no guidelines or regulations or legislations on research ethics [13].

So, it is clear that regulations of clinical trials vary between the countries in the developing world. While some have regulations, others, such as Lebanon, are still developing their laws and guidelines. Yet, Pharmacy Law number 367 dated 11 August 1994 stated indirectly that ‘only teaching hospitals in Lebanon are allowed to conduct clinical research involved with using medications under the Article 55’ [10]. Thus, specific data and deeper analysis of RECs in Lebanon and their adherence to the ethics surrounding conducting clinical trials are required. In this sense, this research project would likely complement the results of the self-assessment project performed within SHBPP. It would also allow elaboration of the recommendations of the Working Party for a more ethical propriety of clinical research in Lebanon. Such results and recommendations would set out the context to train more efficiently the members of the RECs in Lebanon, monitor their development, and avoid conflicts of interests to ensure a strong ethical review of studies carried out in the country.

### Implications of the findings

While regulations of clinical trials are still being developed in Lebanon and one relevant Minister Decree has been released in 2014 (Minister Decree No. 1159/1 of 23 June 2014) [12], Minister Decision No. 141 of 27 January 2016 describes the procedures required from Lebanese RECs to become officially authorised [31]. According to this decision, no clinical study conducted among human subjects can be performed in the country before being reviewed and approved by a REC or an Institutional Review Board (IRB) or Independent Ethics Committee (IEC). Thus, all Lebanese RECs, IRBs or IECs willing to oversee and review the ethical aspects of research that involve human subjects must be registered and officially authorised by the Lebanese Ministry of Public Health [31]. Hospitals and centres that do not have their own RECs are also allowed to be affiliated with authorised RECs per the Ministry’s requirements (Article 7) [31]. This decision also states the guidelines and principles that should be considered by the RECs, namely the International Conference on Harmonisation – Good Clinical Practice, the Standards and Operational Guidance for Ethics Review of Health-related Research with Human Participants’ of the WHO (Article 4) [31], in addition to the ethical principles of the Declaration of Helsinki and Belmont Report (title page) [31]. To be authorised, the REC must submit to the Ministry of Public Health: (1) the REC objectives and working procedures, (2) the membership selection and titles of the REC members, (3) the membership duration, (4) the guidelines used to issue the REC decision, (5) the list of documents required by the REC for a new or ongoing clinical study, to be submitted by the sponsor or its representative, (6) the meeting procedure, and (7) the documents clarifying the voting system, procedure for issuing and reporting the final decision (Article 6) [31]. Following the submission, an IRB evaluation report is completed by the Ministry to allow, or not, the authorisation of the REC/IRB [32] without directly assessing the adherence of the Lebanese RECs to the ethics surrounding the conduct of clinical trials, i.e. the ‘Standards and Operational Guidance for Ethics Review of Health-related Research with Human Participants’ [14].

To counterbalance this gap, our study evaluated the adherence of 10 Lebanese RECs to WHO ethical standards and guidance based on a self-assessment tool developed by Sleem et al. in 2010. Our results showed a total mean score of 129.6 ± 22.3 points out of 175 points, calculated from a sample of 39 participants (all types of members: chairs, vice-presidents, and ordinary members), reflecting thus an excellent adherence to chapters II (standards 2 to 6) and IV (standard 9) of the WHO guidelines. This score tended to be lower compared to the total mean score calculated from the four participating RECs chairs (134.5 ± 15.8 points over 175 points), and higher than the score assessed from the only vice-president who participated in the study (124 over 175 points). In their study conducted in 2011 in a sample of 36 presidents of REC chairs from different developing countries (India, Middle East, South Africa, Central and Eastern Europe), Sleem et al. reported a total mean score of 146.2 ± 34.1 points out of 200 points, reflecting also an excellent adherence to ethical guidelines. After extrapolating our total mean scores over 200 points instead of 175, the total mean scores from all the 39 participants and the four chairs would be 148.1 and 153.7 points, respectively, which tend both to be slightly higher than the results of the regional study [23].

As an aside, all participating RECs (*n* = 10) were reported to be localised in the regions of Beirut and Mount Lebanon. Indeed, all authorised RECs and RECs in the process of approval in Lebanon are localised in these two regions in addition to one REC in South Lebanon, and none are localised in the North or Bekaa. This observation has two possible implications for the conduct clinical trials in Lebanon. First, hospitals and centres localised in the North and Bekaa regions do not have their own RECs implying that no clinical studies are conducted there. A second possibility is that studies are conducted in these hospitals after obtaining REC approval since they are allowed to be affiliated with authorised RECs per the Ministry’s requirements [31].

Furthermore, our main findings show that all Lebanese RECs are mainly composed of individuals with a scientific background (92.3%) followed by non-scientific background (7.7%), which is consistent with standard 2 of chapter II in the WHO guidelines [14]. Also, not all members are affiliated with the REC’s institution which is considered to enhance the REC’s independence. Surprisingly, 10% of the participants reported not having a previous experience with clinical research and 57.7% hold a diploma or had done a training in medical and/or research ethics. Similar findings were reported in a study conducted among 31 RECs across sub-Saharan Africa, as 92% of the participating RECs expressed their need to be trained on the scientific design of clinical trials [27]. Our finding is quite alarming because all members of RECs should have periodic trainings in basic aspects of research methodology, ethical considerations, and reasoning, similar to WHO guidance in standard 5 of chapter II [14]. Hence, the Ministry of Public Health should be notified about these gaps to better control the training requirements for REC members. Otherwise, doubts about the performance of RECs can be raised: how can a protocol be reviewed if not all the REC members are trained in clinical research and research ethics?

As for the conflicts of interests, 28.2% of the participants did not report the availability of a policy for disclosing and managing potential conflicts of interest for the researchers, and 15.4% reported that a conflict of interests form for the researchers is not required by the REC during the submission of the study protocol. Such findings are not consistent with standard 10 of chapter V in the WHO guidance for researchers [14]. Implementing guidelines or SOPs within the REC to identify, disclose, and manage such conflicts of interests can increase the transparency and liability of clinical studies in Lebanon [33].

Importantly, an anti-bribery and anti-corruption policy is reported to be in place by only 33.3% of the participants. Being trained in anti-bribery and anti-corruption acts, having adequate policies or adapting international anti-bribery and corruption policies, such as the UK Anti-Bribery Act [34] and the American Foreign Corrupt Practices Act (FCPA) [35], are crucial for the effective and transparent functioning of the RECs. Such policies forbid sponsors or persons from bribing the REC members, i.e. from making facilitation payments or offering any valuable items to them to impact any official decision.

### Strength and limitations of the study and lessons learned

The ‘Research Ethics Committee Quality Assurance Self-assessment Tool’ developed in 2010 by Sleem et al. was chosen as a reference for data collection in the present study because it refers to international ethical guidelines and applies most to Lebanese cases where most of the local RECs are in their early stages of development. This questionnaire is also quite shorter compared to other self-assessment tools developed in the USA by the Office of Human Research Protections [36], and in the UK by the Association for the Accreditation of Human Research Protection Programmes [37] and the Strategic Initiative for Developing Capacity in Ethical Review [38]. Thus, the completion of the questionnaire by Sleem et al. has shown to be pragmatic, easy, and feasible by the results of their study. [23]. Besides being lengthy (more than 19 pages), the other questionnaires evaluate elements from an American perspective only, not based on international ethical regulations [36], and/or elements that may not be relevant to the protection of human subjects [36–38]. The use of a modified version of the ‘Research Ethics Committee Quality Assurance Self-assessment Tool’ was driven by two motives. First, the modified version allows the collection of additional data about the RECs’ locations and the academic qualifications and educational backgrounds of their members. Second, using a modified version of the questionnaire in the present study would allow the comparison with the outcomes of the study by Sleem et al. Also, the modified version of the ‘Research Ethics Committee Quality Assurance Self-assessment Tool’ used in the present study is shorter than the original questionnaire. The purpose of shortening the original questionnaire was to make it less time-consuming to fill in and less burdensome by removing too many detailed questions, and to avoid the potential participants’ reluctance to participate in the survey because of its length; thus, a low response rate.

Thus, drawing on the study methodology and response rate, the study recorded a higher response rate (50.5%) compared to the estimated rate (30%). A second strength is that the study was representative of all authorised RECs in Lebanon and reported the outcomes from all members of RECs, contrary to the regional study which only solicited chairs of RECs [23]. In this setting, our study allowed us to spot disparities and inconsistencies of answers between the members of the same REC. Indeed, members of the same REC reported differing years of REC establishment, as well as different answers to questions related to the REC organisational aspects, membership and educational training, and policies referring to review procedures. This observation shows that such aspects are not well known to members of the same REC who need to be better trained and informed about the REC organisational aspects, memberships, and policies to allow better performance and a more effective functioning of the committee. Third, since our study surveyed not only chairs of RECs but also ordinary members, it avoided reliance on responses from chairs and distorting the assessments. This is true as chairs might tend to show a very positive aspect of their REC’s performance by agreeing with all the questionnaire’s items. Such tendency to answer ‘yes’ is called acquiescence bias [39].

In parallel, a major limitation of our study is the use of a self-assessment tool which is considered to lack objectivity and accuracy: participants might have the tendency to under- or over-report. Also, the study provides information on what respondents say they do, not what they actually do in practice. Although using such types of tools is not specific enough to explore the REC adherence to international ethical guidelines, a self-assessment tool can capture a general picture of the RECs’ performance in Lebanon. Notably, we did not display the scores assigned to the questions in the online questionnaire on SurveyMonkey® to avoid any additional reporting bias or acquiescent response bias. As such, the participants will not over-report ‘positive aspects’ to be viewed favourably by the surveyor, and results will be more valid.

As for the lessons learned, this work allowed us to learn more about the Lebanese regulations issued by the Ministry of Public Health (Minister Decision No. 141 of 27 January 2016 and Minister Decree No. 1159/1 of 23 June 2014) as regards RECs and the conduct of clinical trials. It highlighted the gaps that need to be addressed by the stakeholders involved in medical research conducted among human subjects. As for the conduct of the present study per se, a better accrual would be achieved if more time was allocated for data collection. In consequence, the results would describe more accurately the adherence of RECs to the international guidelines in ethics, and the comparability to the regional study of Sleem et al. would be less limited.

### Public health relevance and recommendations

Given the increasing number of clinical studies being performed in Lebanon, the present study confirmed the excellent adherence of Lebanese RECs to WHO ethical guidelines [14], but also identified areas of weakness to be considered. For this purpose, we recommend the following steps:

1. To share the study findings with the RECs in Lebanon, especially the participating RECs, to identify their needs and gaps, and do some corrective actions accordingly. For instance, RECs should strengthen the training requirements for all REC members (cf. recommendations 4 to 8)
2. To make the study results available to the public: publication of the study in a peer-reviewed journal
3. To notify the Lebanese Ministry of Public Health about the training gaps to better control the training requirements for REC members
4. To notify the RECs about the trainings that need the most attention, namely: basic aspects of research methodology, ethical considerations and reasoning, anti-bribery and anti-corruption training
5. That the RECs prepare and release SOPs to identify, disclose, and manage conflicts of interests for researchers to increase the transparency and liability of clinical studies in Lebanon
6. That the RECs shape adequate policies or adapt international anti-bribery and corruption policies to forbid sponsors or persons funding clinical studies from bribing the REC members
7. That the members of each REC be better trained and informed about the REC organisational aspects, memberships, and policies to allow better performance and more effective functioning of the committee
8. That The RECs or institution provide better Internet access

## Conclusions

The number and complexity of research study protocols involving human subjects that are reviewed by RECs is increasing in the developing countries such as Lebanon. This dissertation showed that the adherence of the RECs to the ‘Standards and Operational Guidance for Ethics Review of Health-related Research with Human Participants’ [14] is, overall, excellent. It also provides valuable and useful information on the RECs to parties involved in medical research such as sponsors of clinical trials in Lebanon, investigators, members of RECs, and the Ministry of Public Health. The major caveats identified are inadequate training of the REC members, Internet shortage, and lack of anti-bribery policies. Ultimately, the gaps identified through this study should be addressed by the RECs and the Ministry of Public Health to ensure quality improvement of the RECs, instead of just conducting such studies for theoretical purposes.

## Additional file

Additional file 1: Study Questionnaire - RECs in Lebanon. (DOCX 54 kb)

## Abbreviations

AAHRPP: Association for the Accreditation of Human Research Protection Programmes
CIOMS: Council for International Organisations of Medical Sciences
CITI: Collaborative IRB Training Initiative
CV: Curriculum vitae
FCPA: Foreign Corrupt Practices Act
IEC: Independent Ethics Committee
IRB: Institutional Review Board
MD: Medical doctor
MSc: Master of Science
NIH: National Institute of Health
OHRP: Office of Human Research Protections
PhD: Doctor in Philosophy
Q: Quartile
REC: Research Ethics Committee
SIDCER: Strategic Initiative for Developing Capacity in Ethical Review
SOP: Standard Operating Procedures
UK: United Kingdom
WHO: World Health Organisation
WMA: World Medical Association
