# Supplementary material for: Clinical research ethics review process in Lebanon: efficiency and functions of research ethics committees – results from a descriptive questionnaire-based study
Source: Trials. 2018 Jan 11;19:27. doi: 10.1186/s13063-017-2397-2 (PMC5765668; doi:10.1186/s13063-017-2397-2)
Supplement: Additional file 1: — Study Questionnaire - RECs in Lebanon. (DOCX 54 kb) [file 13063_2017_2397_MOESM1_ESM.docx]

Modified version of Research Ethics Committee (REC) Quality Assurance Self-Assessment Tool (Sleem *et al.*, 2010)

The maximum total number of points is 200. For ‘yes/no’ questions, points are given for a ‘yes’ response. All identifiable data will remain confidential and data will be analysed anonymously.

**Participant N°** |___|___|___|___|___| (do not complete, for internal use)

**GENERAL INFORMATION**

1. REC name: __________________________________________

2. Geographical area of the affiliated hospital:  Beirut  Mount Lebanon  North Lebanon

Beqaa  Nabatieh  South Lebanon

3. Year of REC establishment: |___|___|___|___|

4. Your position:  President/chair of the REC  Vice-president of the REC  Member of the REC

5. Your education background:  Medical  Non-medical/scientific  Non-scientific

6. Your academic qualifications: MD  PhD  Pharm D  MSc  Other, specify: __________

7. Do you have any previous experience with clinical research?  Yes  No

8. Do you hold any diploma/training in medical/research ethics?  Yes  No

9. Are you affiliated with the REC’s institution?  Yes  No

**ORGANISATIONAL ASPECTS (Maximum 54 POINTS)**

1. Is the REC subject to registration with a national authority?  Yes  No **2 points**

2. How often does the REC meet as a full committee to review research studies? (**For meeting frequency equal or greater than once/month, 1 point)**

Once/week  Twice/month  Once/month  Every two months

Other  Has not yet met to review protocol

3. Was the REC established under a high ranking authority (e.g., President’s office, Ministry of Health)?

Yes  No **(5 points)**

4. Does the REC have written Standard Operating Procedures?  Yes  No **5 points**

5. Does the REC have a policy that outlines the process for appointing the REC Chair?  Yes  No **2 points**

6. Which of the following criteria are used to select the Chair of the REC? (Check all that apply.)

Prior training in ethics **(1 point)**  Publication in ethics **(1 point)**

Prior research experience **(1 point)**  Other, please describe: __________________________

7. Does the REC have a policy that describes the process for appointing the members of the REC and details the membership requirements and the terms of appointment?  Yes  No **2 points**

8. Which of the following criteria are used to select REC members? (Check all that apply.)

Prior training in ethics **(1 point)**  Publication in ethics **(1 point)**

Prior research experience **(1 point)**  Other, please describe: _________________________

9. Does the REC have a policy for disclosure and management of potential conflicts of interest for the members of the REC?  Yes  No **5 points**

10. Does the REC have a policy for disclosure and management of potential conflicts of interest for members of the research team?  Yes  No **5 points**

11. Does the REC have a quality improvement (QI) program for itself?  Yes  No **5 points**

If yes, describe what was done in the last year and any changes that were made as a result of the QI program._____________________________________________________________________________

12. Does the institution/organization regularly evaluate the operations of the REC (e.g., budgetary needs, adequacy of material resources, adequacy of policies and procedures and practices, appropriateness of the membership given the research being reviewed, and documentation of the training requirements of the REC members)?

Yes  No **5 points**

13. Does the REC have a mechanism whereby enrolled research participants can file complaints or direct questions regarding human subjects’ protection issues?

Yes  No **5 points**

If yes, please describe the mechanism. ______________________________________________

14. How are records of the REC stored?  **(1 point maximum)**

Paper folders in a locked file cabinet **(1 point)**  Electronic in a password-protected computer **(1 point)**

On an open shelf  Other

15. Quorum: Does the REC require that there be a certain number of members present in order to make the meeting official to review protocols?  Yes  No **5 points**

**CONFLICTS OF INTERESTS / ANTI-BRIBERY / ANTI-CORRUPTION POLICY (0 POINT)**

1. Do you have any anti-bribery / anti-corruption policy within your IEC/IRB?  Yes  No
2. Do you have any policy that define whether the members have to be trained on anti-bribery and anti-corruption course?

Yes  No

- 1. If yes, what is the frequency of training? ___________

1. Do you accept goods, gifts of money or other items of value?  Yes  No
   1. If yes, what is your gifts policy (i.e. when do you accept gifts)?  Yes  No

**MEMBERSHIP AND EDUCATIONAL TRAINING (Maximum 30 POINTS)**

1. How many members are there on the REC? |___|___| **If ≥ 5 members, 2 points**

2. How many are women? |___|___| How many are men? |___|___| **If female/male gender ratio is between 0.4 and 0.6, then 2 points**

3. Are any of the members not affiliated with the institution, that is, the member is not employed by the institution and is not related to a person who is employed?  Yes  No **2 points**

4. Are any of the members considered to be a non-scientist?  Yes  No **2 points**

(A Non-Scientific Member is any member who does not have a terminal degree in a medical or scientific field.)

***Please note that one member may fulfil both criteria of non-scientist and non-affiliated, in which case, please check Yes for both #3 and #4.***

5. Is there a requirement that the REC Chair (or the designee who is in charge of running the committee) has any prior formal training in research ethics?  Yes  No **5 points**

If yes, what type of training is required? (Check all that apply.)

Web-based training  Workshop in research ethics

Course  Other, please describe: _____________________________

6. Does the institution require that REC members have training in research ethics in order to be a member of the REC?  Yes  No **5 points**

If yes, what type of training is required? (Check all that apply.)

Web-based training  Workshop in research ethics

Course  Other, please describe: ______________________________

7. Does the institution require that investigators have training in research ethics to submit protocols for review by the REC?  Yes  No **5 points**

If yes, what type of training is required? (Check all that apply.)

Web-based training  Workshop in research ethics

Lecture  Course

Other, please describe: __________________________________________________________

8. Does the REC conduct continuing education in research ethics for its members on a regular basis?

Yes  No **5 points**

9. Does the REC document the human subjects’ protection training received by its members?

Yes  No **2 points**

**SUBMISSION ARRANGEMENTS AND MATERIALS (Maximum 7 POINTS)**

| **Submission Materials** |  | |
| --- | --- | --- |
| **Which of the following items are requested from the Principal Investigators when they submit their research protocol to the REC:** | **1 point each** | |
| **Item** | **Yes** | **No** |
| Full protocol? |  |  |
| Informed Consent Form? |  |  |
| Investigator’s qualifications [e.g., CV, medical license(s), etc.]? |  |  |
| Conflict of interests disclosure forms for members of the research team? |  |  |
| Recruitment material (e.g., advertisements, signs, posters, etc.), if applicable? |  |  |
| Questionnaires/surveys that will be used in the research, if applicable? |  |  |
| Investigators’ Drug Brochure or materials describing the nature of the drug being used in a clinical trial, if applicable? |  |  |

**POLICIES REFERRING TO REVIEW PROCEDURES (Maximum 9 POINTS)**

| **Policies Referring to Review Procedures** | **1 point each** | |
| --- | --- | --- |
| **Item** | **Yes** | **No** |
| Does the REC have a policy regarding how protocols will be reviewed? |  |  |
| Does the REC bring in a consultant when necessary to provide scientific or other relevant expertise for review of a particular protocol? |  |  |
| Do REC members receive the protocol and other materials at a specified time prior to the meeting? |  |  |
| Does the REC require that reviewers use a checklist to document their ethical assessment of the research submission? |  |  |
| Does the REC determine the interval of continuing review based on the risk of the study? |  |  |
| Does the REC have a policy for how decisions are made (e.g., consensus or a vote)? |  |  |
| Are members asked at the beginning interest regarding any the meeting as to whether they had a conflict of the protocols to be discussed and indicate that such members did not participate in the decision on the relevant protocols? |  |  |
| Does the REC have a policy for communicating a decision? |  |  |
| Does the REC have a policy for follow-up review? |  |  |

**REVIEW OF SPECIFIC PROTOCOL ITEMS (Maximum 38 POINTS)**

| **Scientific Design and Conduct of the Study** | **1 point each** | |
| --- | --- | --- |
| **Item** | **Yes** | **No** |
| Does the REC review the suitability of the investigators’ qualifications to conduct the study? |  |  |
| Does the REC review the adequacy of the clinical site, including the supporting staff, available facilities, and emergency procedures? |  |  |

| **Considerations of Risks and Benefits** | **1 point each** | |
| --- | --- | --- |
| **Item** | **Yes** | **No** |
| Does the REC identify the different risks of the research protocol? |  |  |
| Does the REC determine whether risks have been minimized? |  |  |
| Does the REC determine whether the risks are greater than minimal risk based on a written definition of minimal risk? |  |  |
| Does the REC evaluate the probable benefits of the research to the participants? |  |  |
| Does the REC evaluate whether the risks to research participants are reasonable in relation to any anticipated benefits to participants and the importance of the knowledge to be gained by society? |  |  |

| **Selection of Research Participants** | **1 point each** | |
| --- | --- | --- |
| **Item** | **Yes** | **No** |
| Does the REC review the methods to identify and recruit potential participants? |  |  |
| Does the REC identify the potential of the research for enrolling participants who are likely to be vulnerable to coercion or undue influence (such as children, prisoners, persons with mental disabilities, or persons who are economically or educationally disadvantaged)? |  |  |
| Does the REC consider the justification for including vulnerable populations in the research? |  |  |
| Does the REC consider and require that additional safeguards be included in the study to protect the rights and welfare of the subjects? |  |  |
| Does the REC consider the appropriateness of any financial or material incentives offered to participants for their participation in the research? |  |  |

| **Privacy and Confidentiality** | **1 point each** | |
| --- | --- | --- |
| **Item** | **Yes** | **No** |
| Does the REC preserve privacy by evaluating the setting in which participants are recruited? |  |  |
| Does the REC evaluate the methods for protecting the confidentiality of the collected research data? |  |  |

| **Community Consultation** | **1 point each** | |
| --- | --- | --- |
| **Item** | **Yes** | **No** |
| Does the REC review whether the potential benefits of the research are relevant to the health needs of the local community/country? |  |  |

| **Safety Monitoring and Adequacy of Insurance to Cover Research-Related Injury** | **1 point each** | |
| --- | --- | --- |
| **Item** | **Yes** | **No** |
| Does the REC require, when appropriate, that the research plan include adequate provisions for monitoring the data collected to ensure the safety of subjects? |  |  |
| Does the REC consider whether the sponsors of the research have adequate insurance to cover the treatments of injury related to the research? |  |  |

| **Paediatric Research** | **1 point each** | |
| --- | --- | --- |
| **Item** | **Yes** | **No** |
| Does the REC evaluate the need to obtain the child’s assent? |  |  |

| **Informed Consent** | **1 point each** | |
| --- | --- | --- |
| **Item** | **Yes** | **No** |
| Does the REC review the process by which informed consent will be obtained (e.g., how do investigators identify potential subjects, where does the informed consent process take place, are potential subjects allowed to take the consent form home and given enough time to ask questions, etc.)? |  |  |
| Does the REC review which members of the research team will approach potential participants for their informed consent? |  |  |
| Does the REC ensure that the informed consent document is understandable to the subject population?  Suggested ways to assess the consent form might include:  • evaluate the reading level of the consent document  • have a community member read the consent form  • require investigators to assess subjects’ understanding of the consent form |  |  |
| Does the REC waive the requirement to obtain informed consent that is based on written criteria? |  |  |
| Does the REC waive the requirement to have a written signature on the informed consent document that is based on written criteria? |  |  |

| **Basic Elements of Informed Consent** |  | |
| --- | --- | --- |
| **Does the REC evaluate whether informed consent forms contain the following basic elements of informed consent:** | **1 point each** | |
| **Item** | **Yes** | **No** |
| A statement that the study involves research? |  |  |
| An explanation of the purposes of the research? |  |  |
| The expected duration of the subject’s participation? |  |  |
| A description of the procedures to be followed? |  |  |
| Identification of any experimental procedures? |  |  |
| A description of any reasonably foreseeable risks or discomforts to the participant? |  |  |
| A description of any benefits to the participant or to others that might reasonably be expected from the research? |  |  |
| A disclosure of appropriate alternative procedures or courses of treatment, if any, that might be advantageous to the subject? |  |  |
| A statement describing the extent, if any, to which confidentiality of records identifying the participant will be maintained? |  |  |
| For research involving more than minimal risk, an explanation as to whether any medical treatments are available if injury occurs and, if so, what the treatments consist of or where further information may be obtained? |  |  |
| An explanation of whom to contact for answers to pertinent questions about research |  |  |
| An explanation of whom to contact for answers to pertinent questions about research participants’ rights |  |  |
| A statement that participation is voluntary |  |  |
| A statement that refusal to participate will involve no penalty or loss of benefits to which the subject is otherwise entitled |  |  |
| A statement that participant may discontinue participation at any time without penalty or loss of benefits to which the participant is otherwise entitled |  |  |

**COMMUNICATING A DECISION (APPROVAL LETTER) (Maximum 5 POINTS)**

Please answer the following questions regarding the approval letter sent to the PI. If no approval letter is sent to the investigator, please **skip** this section.

| **Which of the following items are in the approval letter?** | **1 point each** | |
| --- | --- | --- |
| **Item** | **Yes** | **No** |
| Provide an expiration date that is 1 year from the date of the convened REC meeting in which the study was approved. |  |  |
| Require the investigators to submit to the REC as an amendment any changes that occur in the research plan; for example, change in investigators, change in drug doses, change in the sample size, etc. |  |  |
| Require the investigators to promptly report to the REC any adverse events or unanticipated problems |  |  |
| Require the investigators to promptly report to the REC any protocol deviations. |  |  |
| Require investigators to use the REC-approved informed consent form that is stamped with an expiration date. |  |  |

**CONTINUING REVIEW (Maximum 16 POINTS)**

| **Does the REC request a continuing review report from the investigators on at least a yearly basis?**  **Yes**  **No** | **5 points** | |
| --- | --- | --- |
| **If yes,** which of the following items are requested in the continuing review report? | **1 point each** | |
| **Item** | **Yes** | **No** |
| Number of subjects enrolled |  |  |
| Gender and ethnic/religious breakdown of enrolled subjects |  |  |
| Number of subjects withdrawn from the research by the investigators |  |  |
| The reasons for withdrawal |  |  |
| Number of subjects who dropped out of the research |  |  |
| The reasons why subjects dropped out |  |  |
| Verification that informed consent was obtained from all subjects and that all signed consent forms are on file |  |  |
| Number and description of serious adverse events in the previous year (SAEs) |  |  |
| List of any protocol violations or deviations |  |  |
| Any safety monitoring reports |  |  |
| If the study is completed, submit a final report describing the study results |  |  |

**REC RESOURCES (Maximum 16 POINTS)**

1. Does the REC(s) have its own yearly budget?  Yes  No **5 points**

If yes, is there a budget for training of administrative staff and REC members?  Yes  No **1 point**

2. Please check below the physical resources of the REC (check all that apply): **1 point each**

Access to a meeting room  Access to a computer and printer  Access to the Internet

Access to a facsimile  Access to cabinets for storage of the protocol files

3. Does the REC have administrative staff assigned to the REC?  Yes  No **5 points**

**Thank you for your time and participation.**

## Appendix 4: Review of specific protocol items by the REC (N=39)

|  | **Number**  **(n)** | **Percentage**  **(%)** |
| --- | --- | --- |
| **Scientific Design and Conduct of the Study** | | |
| Review of the suitability of the investigators’ qualifications to conduct the study | 28 | 71.8 |
| Review of the adequacy of the clinical site, including the supporting staff, available facilities, and emergency procedures | 31 | 79.5 |
| **Considerations of Risks and Benefits** | | |
| Identification of the different risks of the research protocol | 37 | 94.9 |
| Determination of whether risks have been minimized | 37 | 94.9 |
| Determination of whether the risks are greater than minimal risk based on a written definition of minimal risk | 36 | 92.3 |
| Evaluation of the probable benefits of the research to the participants | 38 | 97.4 |
| Evaluation of whether the risks to research participants are reasonable in relation to any anticipated benefits to participants and the importance of the knowledge to be gained by society | 38 | 97.4 |
| **Selection of Research Participants** | | |
| Review of the methods to identify and recruit potential participants | 38 | 97.4 |
| Identification of the potential of the research for enrolling participants who are likely to be vulnerable to coercion or undue influence | 36 | 92.3 |
| Consideration of the justification for including vulnerable populations in the research | 37 | 94.9 |
| Considerations and requirements of additional safeguards to be included in the study to protect the rights and welfare of the subjects | 38 | 97.4 |
| Consideration of the appropriateness of any financial or material incentives offered to participants for their participation in the research? | 37 | 94.9 |
| **Privacy and Confidentiality** | | |
| Preservation of the privacy by evaluating the setting in which participants are recruited | 38 | 97.4 |
| Evaluation of the methods for protecting the confidentiality of the collected research data | 36 | 92.3 |
| **Community Consultation** | | |
| Review of whether the potential benefits of the research are relevant to the health needs of the local community/country | 35 | 89.7 |
| **Safety Monitoring and Adequacy of Insurance to Cover Research-Related Injury** | | |
| Requirement, when appropriate, that the research plan include adequate provisions for monitoring the data collected to ensure the safety of subjects | 37 | 94.9 |
| Consideration of whether the sponsors of the research have adequate insurance to cover the treatments of injury related to the research | 37 | 94.9 |
| **Paediatric Research** | | |
| Evaluation of the need to obtain the child’s assent | 36 | 92.3 |
| **Informed Consent** | | |
| Review of the process by which informed consent will be obtained | 38 | 97.4 |
| Review of which members of the research team will approach potential participants for their informed consent | 37 | 87.2 |
| Insurance that the informed consent document is understandable to the subject population (evaluation of the reading level of the consent document, having a community member read the consent form, and requiring investigators to assess subjects’ understanding of the consent form) | 35 | 89.7 |
| Waiver of the requirement to obtain informed consent that is based on written criteria | 27 | 69.2 |
| Waiver of the requirement to have a written signature on the informed consent document that is based on written criteria | 28 | 71.8 |
| **Basic Elements of Informed Consent: the REC evaluates whether informed consent forms contain the following basic elements of informed consent:** | | |
| A statement that the study involves research | 39 | 100 |
| An explanation of the purposes of the research | 39 | 100 |
| The expected duration of the subject’s participation | 39 | 100 |
| A description of the procedures to be followed? | 39 | 100 |
| Identification of any experimental procedures | 39 | 100 |
| A description of any reasonably foreseeable risks or discomforts to the participant | 39 | 100 |
| A description of any benefits to the participant or to others that might reasonably be expected from the research | 39 | 100 |
| A disclosure of appropriate alternative procedures or courses of treatment, if any, that might be advantageous to the subject | 38 | 97.4 |
| A statement describing the extent, if any, to which confidentiality of records identifying the participant will be maintained | 38 | 97.4 |
| For research involving more than minimal risk, an explanation as to whether any medical treatments are available if injury occurs and, if so, what the treatments consist of or where further information may be obtained | 37 | 94.9 |
| An explanation of whom to contact for answers to pertinent questions about research | 38 | 97.4 |
| An explanation of whom to contact for answers to pertinent questions about research participants’ rights | 38 | 97.4 |
| A statement that participation is voluntary | 39 | 100 |
| A statement that refusal to participate will involve no penalty or loss of benefits to which the subject is otherwise entitled | 38 | 97.4 |
| A statement that participant may discontinue participation at any time without penalty or loss of benefits to which the participant is otherwise entitled | 39 | 100 |
